# Cancer-associated fusion transcripts: mechanisms, functional roles, and clinical implications

**DOI:** 10.1007/s10238-026-02303-3

**Published:** 2026-08-31

**Authors:** Md Faruq Hossain

**Affiliations:** Human Molecular Genetics Group, Department of Functional Genomics, Interfaculty Institute for Genetics and Functional Genomics, University Medicine Greifswald, Greifswald, Germany

**Keywords:** Fusion transcripts, Cancer biomarkers, Chromosomal rearrangements, Artificial intelligence, CRISPR/Cas9, Precision oncology

## Abstract

Fusion transcripts are hybrid RNA molecules generated through genomic rearrangements or RNA-level fusion mechanisms. They represent important molecular features of many cancers and can function as oncogenic drivers, diagnostic biomarkers, prognostic indicators, and therapeutic targets. Since the discovery of the BCR::ABL1 fusion in chronic myeloid leukemia, numerous cancer-associated fusion transcripts have been identified across hematologic malignancies and solid tumors. These fusion events encompass diverse biological mechanisms, including constitutively active kinases, aberrant transcription factors, epigenetic regulators, and non-coding fusion RNAs. This review summarizes current knowledge of the mechanisms underlying fusion transcript formation, including genomic rearrangement-dependent and rearrangement-independent processes, as well as fusion circular RNAs. The functional roles of fusion transcripts in cancer biology and their clinical relevance as diagnostic, prognostic, and predictive biomarkers are discussed. In addition, recent advances in fusion transcript detection and characterization are reviewed, including next-generation sequencing, long-read sequencing, single-cell approaches, artificial intelligence-assisted computational methods, and CRISPR/Cas9-mediated strategies for functional modeling and functional validation of fusion transcripts. Despite the rapid expansion of fusion transcript catalogs, the biological and clinical significance of most identified fusion events remains incompletely understood. Future progress will depend on integrating advanced sequencing technologies, artificial intelligence-assisted computational prioritization, and systematic functional validation to distinguish clinically actionable fusion transcripts from biologically neutral events. Such multidisciplinary approaches will be essential for translating fusion transcript research into precision oncology and improving cancer diagnosis, patient stratification, and targeted therapy.

## Introduction

Fusion transcripts (FTs), often also termed fusion RNAs or chimeric transcripts, are hybrid mRNAs that are often associated with oncogenic processes in cancer. To date more than 23,000 FTs, which are linked to cancer, are reported in the ChiTaRS database [1] while the Mitelman Database of Chromosome Aberrations and Gene Fusions contains around 34,718 unique fusion genes that are associated with cancer [2, 3]. In addition, comprehensive cancer knowledgebases such as the Catalogue of Somatic Mutations in Cancer (COSMIC) and OncoKB provide complementary information by integrating genomic, functional, and clinical evidence for recurrent fusion events, including their prevalence, oncogenic significance, and therapeutic actionability. Although these resources differ in their scope, curation strategies, and evidence criteria, together they represent invaluable tools for fusion discovery, annotation, and precision oncology [4, 5]. As these databases are continuously curated and expanded, the reported numbers of fusion events and associated genes may change over time. FTs are mainly derived from fusion genes, which arise from structural chromosomal rearrangements (see below).

FTs represent a heterogeneous group of molecular events with varying functional consequences, ranging from strong oncogenic drivers to biologically neutral byproducts. FTs can have a variety of effects on gene expression and protein function. In some cases, the FT may result in the production of a novel protein with altered or enhanced activity compared to the protein products of the original genes involved in the fusion. In other cases, an FT can result in the loss of function of one or both of the genes of origin (see below). FTs can be used as diagnostic or prognostic markers for certain types of cancer, as they are often associated with specific cancer subtypes. A systematic investigation of gene fusions in 9,624 tumors across 33 cancer types was performed by Gao et al. in 2018 using multiple fusion-calling tools [6]. This study indicated that fusion events play a significant role in 16.5% of human cancers, with over 1% of cases being solely driven by fusions [6]. These fusion events can contribute to oncogenicity by altering the expression of tumor suppressors or proto-oncogenes. FTs may interfere with the cellular proteome by encoding fusion proteins, thereby promoting tumorigenesis. Notably, some fusion proteins can constitute immunogenic neoantigens, making them potential targets for personalized immunotherapy. Fusion RNAs are also found in normal cells and tissues and not all FTs are linked to cancer [7]. These RNAs may contribute to cellular regulatory processes.

The discovery of FTs has greatly expanded our understanding of the genetic basis of cancer and other diseases. By identifying and characterizing FTs, it is possible to gain insights into the molecular mechanisms underlying disease development and progression, and develop new diagnostic as well as therapeutic strategies. Recent advances in computational approaches, particularly artificial intelligence and machine learning, have further expanded the ability to detect and interpret FTs at scale. Earlier reviews and most individual studies have primarily focused on the identification, detection, and diagnosis of different FTs, and the role of linear and/or circular fusion RNAs in individual cancers, as they usually deal with common fusions or specific cancer types. In this review, I provide an inclusive overview where I systematically organize and classify known FTs associated with different cancers and their functions. In addition, I address advancements concerning the detection tools, validation, and characterization of those fusions. Finally, I highlight the role of FTs as diagnostic and prognostic biomarkers and summarize state-of-the-art in vitro and in vivo methods, including CRISPR/Cas9 based approaches, to generate cancer-associated FTs.

The following sections first summarize the molecular mechanisms of FT formation, followed by their biological functions, technological advances in detection and modeling, computational approaches, clinical applications, therapeutic targeting, and future perspectives.

## Mechanisms of fusion transcript formation

FTs most commonly arise from genomic rearrangements involving genes located on the same or different chromosomes and can be generated through mechanisms such as translocations, insertions, inversions, deletions, and tandem duplications. The formation of such genomic rearrangements is primarily driven by DNA double-strand breaks and their repair through pathways such as non-homologous end joining (NHEJ), microhomology-mediated end joining (MMEJ), and homologous recombination (HR) [8]. In addition, the three-dimensional organization of the genome contributes to fusion formation, as spatial proximity of genomic loci within the nucleus increases the likelihood of recurrent rearrangements. Erroneous DNA repair of these lesions can then lead to the generation of gene fusions, resulting in FTs after transcription [9].

Although FTs arise through somatic genomic rearrangements, inherited genetic variation may influence an individual’s susceptibility to cancers characterized by specific fusion events. A prominent example of this relationship occurs in ETV6::RUNX1-positive childhood acute lymphoblastic leukemia. Genome-wide association studies (GWAS) identified the germline variant rs17481869 at 2q22.3 as a susceptibility locus for ETV6::RUNX1-positive B-cell precursor ALL, while a subsequent study identified rs10853104 within IGF2BP1 as a subtype-specific risk variant [10, 11]. Particularly, rs10853104 is located within a transcription-factor-binding region and is predicted to disrupt a conserved CTCF-binding motif, providing a potential link between inherited variation, chromatin organization, and susceptibility to the fusion-positive leukemia subtype [11]. Evidence for germline somatic interactions has also been reported in prostate cancer. Germline susceptibility loci show differential associations with TMPRSS2::ERG-positive and fusion-negative tumors, while variants within the HNF1B locus have been associated with fusion-status-dependent regulatory effects [12]. However, these associations do not demonstrate that individual germline SNPs directly induce DNA double-strand breaks or fusion formation. Rather, they suggest that inherited genomic background may modify susceptibility to particular fusion-defined cancer subtypes, while the mechanisms directly linking specific germline variants to somatic rearrangements remain incompletely understood.

Beyond inherited genetic variation, epigenetic regulation can influence the formation of genomic rearrangements and thereby contribute to fusion generation. Direct experimental evidence has demonstrated such a mechanism at the KMT2A (formerly MLL) locus. The balance of histone H3 lysine 9 mono- and dimethylation (H3K9me1/2) at this locus is regulated through the opposing activities of the lysine demethylase KDM3B and the methyltransferase G9a/EHMT2. Depletion of KDM3B increases H3K9me1/2 and reduces CTCF occupancy at the KMT2A locus, thereby promoting KMT2A amplification and genomic rearrangements. Conversely, inhibition of G9a suppresses these alterations, including KMT2A rearrangements induced by doxorubicin, providing direct evidence that chromatin state can influence the susceptibility of specific genomic loci to structural alteration [13].

Chronological age may also influence the occurrence of chromosomal rearrangements. An international pooled analysis of 1,933 healthy individuals demonstrated that chromosome translocation frequencies in peripheral blood lymphocytes increase significantly with age, with an accelerated increase observed particularly after approximately 60 years of age [14]. Several biological echanisms may contribute to this age-associated increase. DNA damage accumulates with age, while the efficiency and fidelity of DNA double-strand break repair, including non-homologous end joining and homologous recombination, may decline during aging [15, 16]. In addition, stable chromosomal translocations can persist through mitosis, allowing affected cells and their progeny to remain viable and thereby contributing to their accumulation over time. Environmental and lifestyle-related genotoxic exposures may further contribute to this process; notably, smoking was associated with increased translocation frequency and a steeper age-related increase in the same pooled analysis [14]. However, the mechanisms responsible for the increased frequency of chromosomal rearrangements at older ages remain incompletely understood. Together, these findings indicate that FT formation is influenced not only by DNA damage and repair mechanisms but also by inherited susceptibility, epigenetic regulation, and age-related genomic instability.

Although genomic rearrangements represent the predominant mechanism of FT formation, not all FTs arise directly from structural alterations at the DNA level. Some FTs are generated through RNA-level mechanisms, including read-through transcription and cis- or trans-splicing. In addition, certain genomic rearrangements do not create chimeric coding sequences but instead activate oncogenes through enhancer hijacking or promoter swapping. The following subsections therefore distinguish genomic rearrangement-dependent, genomic rearrangement-independent, and circular RNA-based mechanisms of FT formation.

### Genomic rearrangement dependent mechanisms

**Fig. 1:**
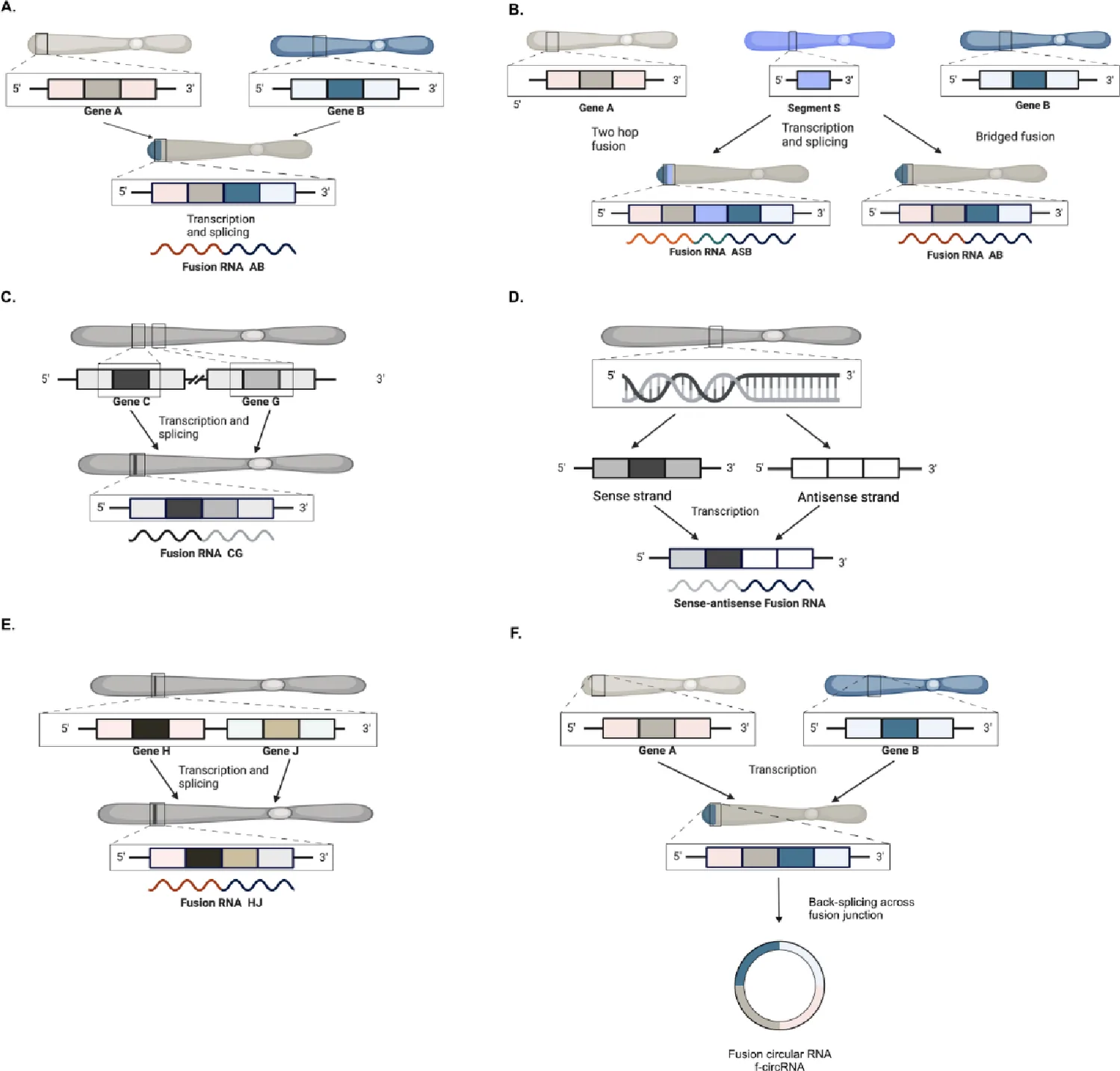
Mechanisms underlying the formation of cancer-associated fusion transcripts. (**A**) Genomic rearrangement-dependent fusion formation through chromosomal translocation, where DNA double-strand breaks and abnormal repair bring together segments from two different genes, generating a fusion gene that can produce a chimeric transcript. (**B**) Additional DNA-level mechanisms contributing to fusion formation, including inversions, insertions, deletions, and tandem duplications, which can alter gene structure and generate FTs after transcription. (**C**) RNA-level fusion formation through trans-splicing, in which exons from two independent pre-mRNA molecules are joined to generate a chimeric RNA without an underlying DNA rearrangement. (**D**) Formation of sense-antisense (SAS) FTs through the joining of transcripts derived from opposite strands of the same genomic region, which may function as regulatory RNAs or influence expression of parental genes. (**E**) Cis-splicing between adjacent genes (cis-SAGe), where transcriptional read-through and splicing between neighboring genes generate FTs while the genomic structure remains intact. (**F**) Formation of fusion circular RNAs (f-circRNAs) through back-splicing across fusion junctions generated by chromosomal rearrangements, resulting in stable circular RNA molecules with potential regulatory and oncogenic functions

FTs typically originate from genomic structural alterations caused by DNA damage and subsequent erroneous recombination and replication. Replication-based mechanisms, including fork stalling and template switching (FoSTeS) as well as break-induced replication (BIR), may further contribute to complex rearrangement patterns observed in multi-segment fusions [17]. Depending on a number of structural rearrangement events that occur during the original fusion event, genomic rearrangement dependent fusions can be subdivided into two categories: single- and multi-segment fusions. Single-segment gene fusions involve the exchange of segments between two genomic loci, often located on non-homologous chromosomes. Generally, exons from two distinct genomic loci become juxtaposed and are transcribed as a FT (Fig. 1A). Multi-segment gene fusions involve the combination of multiple segments or exons from multiple genes. Within this fusion category, two fusion subtypes can be distinguished, namely (i) the “Two Hop Fusion” and (ii) the “Bridged Fusion” (Fig. 1B). The Two Hop Fusion subtype involves an additional segment from a third genomic location that bridges two genes. Fusions generated through this mechanism retain the genomic region from the third segment in their mature RNA transcripts [18]. The inserted genomic segment may contribute additional sequence to the mature transcript and thereby influence its structure and function. These inserted sequences may undergo additional splicing and can influence the structure and functional properties of the resulting FT [19].

The second subtype, “Bridged Fusion”, also requires a third genomic location to bridge two genes. Unlike the Two Hop Fusion subtype, mature RNA transcripts generated through bridged fusion typically do not retain the bridging sequence. A special category of genomic rearrangement–dependent event comprises intragenic FTs, which arise when structural alterations such as deletions, tandem duplications, inversions, or internal rearrangements occur within a single gene locus. Unlike classical intergenic fusions, these events involve the joining of different segments of the same gene and can result in truncated proteins, exon-skipping variants, or proteins with altered functional properties. In several cases, intragenic FTs act as oncogenic drivers by disrupting normal protein regulation or generating constitutively active protein variants. Representative examples include ERG::ERG fusion, FOXO1::FOXO1, EGFR::EGFR (EGFRvIII), AMACR::AMACR, PDGFRA::PDGFRA, RET::RET, and ALK::ALK fusions, which have been reported in a variety of cancer types [19–21].

### Genomic rearrangement independent mechanisms

Genomic rearrangement independent fusion mechanisms involve multiple processes at the RNA level. Compared with genomic rearrangement-dependent mechanisms, these events occur without detectable DNA-level breakpoints, and the underlying genomic structure remains intact. RNA-level FTs may arise through dynamic processes influenced by transcriptional activity, splicing regulation, and cellular context. Trans-splicing and cis-splicing between adjacent genes (cis-SAGe) represent the major mechanisms within this category.

Trans-splicing is the joining of exons from two different pre-mRNA molecules. These processes can be influenced by transcriptional read-through, inefficient transcription termination, or dysregulation of the splicing machinery, particularly under conditions of cellular stress or epigenetic alteration. Trans-splicing can occur between transcripts derived from neighboring genes, distant loci on the same chromosome, genes located on different chromosomes, or distinct transcripts of the same gene (Fig. 1C). Several cancer-associated RNA-level FTs have been experimentally validated using RT-PCR and sequencing approaches, including SLC45A3::ELK4 in prostate cancer, which represents a recurrent FT generated independently of a detectable chromosomal rearrangement [22].

A subset of intragenic trans-splicing events gives rise to sense-antisense (SAS) fusions, which arise from the fusion of bidirectional transcripts originating from the same gene. Generally, SAS fusions are tissue specific although some are recurrent across different tissue types. Several SAS fusion RNAs have been proposed to regulate gene expression by acting as long non-coding RNAs (lncRNAs) and interacting with their parental transcripts (Fig. 1D).

Cis-splicing between adjacent genes (cis-SAGe) involves the splicing of exons from two adjacent genes on the same chromosome. This splicing occurs when two genes are in close proximity, which allows the transcription of one gene to extend into the neighboring gene. In such cases the splicing machinery recognizes splice sites from both genes (Fig. 1E). Several cis-SAGe transcripts have been experimentally confirmed, including fusion RNAs identified in prostate cells, supporting the existence of RNA-level fusion mechanisms independent of DNA rearrangement [23].

Interestingly, not all FTs identified in cancer are strictly cancer-specific. KANSL1::ARL17A and KANSL1::ARL17B are examples of FTs associated with the common 17q21.31 inversion polymorphism [24]. Although these transcripts have been reported in certain malignancies, including T-cell lymphoblastic lymphoma (T-LBL) and medulloblastoma, they are also expressed in non-cancerous tissues, indicating that FT formation can also occur as part of normal transcriptional processes [25, 26]. This observation highlights that the presence of a FT alone does not necessarily imply oncogenic function and underscores the importance of functional characterization when evaluating the biological and clinical relevance of newly identified fusion events.

### Fusion circular RNAs

Fusion circular RNAs (f-circRNAs) are a subset of circular RNAs that derive from cancer-associated chromosomal translocations [27]. They are produced when back-splicing occurs across the fusion junction formed between rearranged partner genes (Fig. 1F) [28]. Generally, f-circRNAs are more stable than linear RNAs because of their covalently closed loop structure lacking free 5′ and 3′ ends [29]. Functionally, f-circRNAs have been shown to act as microRNA sponges, interact with RNA-binding proteins, and in some cases contribute to tumor progression and therapy resistance [30].

Beyond their biological functions, f-circRNAs have also emerged as potential therapeutic targets. Their covalently closed structure confers remarkable stability, making them attractive biomarkers for liquid biopsy and disease monitoring. Moreover, their unique fusion junctions provide highly specific targets for antisense oligonucleotides, RNA interference, and CRISPR/Cas13-mediated RNA degradation [31, 32]. Although these approaches remain largely at the preclinical stage, f-circRNAs represent a promising class of molecules for future precision oncology strategies.

Taken together, these observations highlight that FT formation is not a uniform process but arises from multiple genomic and transcriptomic mechanisms, the relative contributions of which may vary across cancer types and cellular contexts.

## Cancer-associated fusion transcripts and their functional roles

The identification of cancer-associated FTs has expanded substantially over the past decades, driven by continuous advances in cytogenetic and sequencing technologies. Early observations of recurrent chromosomal abnormalities provided the first evidence that structural genome alterations can contribute to tumorigenesis. A landmark discovery was made in 1960 by Peter Nowell and David Hungerford, who described a recurrent abnormal chromosome in patients with CML, later termed the Philadelphia chromosome [33, 34]. This aberration was subsequently shown by Janet Rowley to arise from a reciprocal translocation t(9;22)(q34;q11), generating the oncogenic fusion gene BCR-ABL1 [35]. This finding provided the first direct link between chromosomal rearrangements, FTs, and cancer pathogenesis.

With the development of chromosome banding techniques in the early 1970s, additional recurrent and disease-specific translocations were identified across hematologic malignancies [36]. Many of these rearrangements were later shown to produce FTs that function as key oncogenic drivers. For example, the translocation t(15;17)(q22;q21) in acute promyelocytic leukemia (APL) generates the PML::RARA transcript, which disrupts retinoic acid receptor signaling and blocks myeloid differentiation [37].While PML::RARA represents the defining molecular lesion of classical APL, atypical retinoic acid receptor rearrangements with distinct biological and therapeutic properties have also been identified. A recent example is the tripartite PML::RARG::LINE-L2a fusion in RARG-related atypical APL. The incorporation of LINE-L2a sequence results in truncation of the RARG ligand-binding domain and contributes to all-trans retinoic acid (ATRA) unresponsiveness. Interestingly, despite resistance to ATRA, the reported PML::RARG::LINE-L2a case showed sensitivity to arsenic-based therapy, distinguishing it from other RARG-rearranged atypical APLs that are generally resistant to both ATRA and arsenic trioxide (ATO). This finding illustrates how differences in fusion architecture can influence therapeutic response and highlights the importance of precise molecular characterization of APL-like malignancies [38, 39]. Similarly, the t(8;21)(q22;q22) translocation in acute myeloid leukemia produces RUNX1::RUNX1T1, altering transcriptional programs essential for hematopoietic differentiation [40]. In Burkitt lymphoma (BL), translocations involving the MYC locus, such as t(8;14)(q24;q32), result in deregulated MYC expression through juxtaposition with immunoglobulin enhancers, highlighting how chromosomal rearrangements can drive oncogenesis even in the absence of chimeric coding sequences [41].

Subsequent studies revealed that FTs are not restricted to hematologic cancers but are also highly prevalent and often disease-defining in solid tumors, particularly those of mesenchymal origin. A well-characterized example is the EWSR1::FLI1 transcript generated by t(11;22)(q24;q12) in Ewing sarcoma, which acts as an aberrant transcription factor driving oncogenic gene expression programs [42]. Similarly, PAX3::FOXO1 in alveolar rhabdomyosarcoma (ARMS) and FUS::DDIT3 in myxoid liposarcoma exemplify fusion-driven transcriptional rewiring in solid tumors [43, 44].

Importantly, recurrent fusion events have also been identified in benign tumors, such as HMGA2-related rearrangements in lipomas, indicating that FTs can contribute to both malignant and non-malignant proliferative processes [45].

Functionally, cancer-associated FTs can be broadly grouped into several mechanistic classes. A major category includes constitutively active kinases, exemplified by BCR::ABL1, EML4::ALK, and KIF5B::RET, which drive persistent activation of downstream signaling pathways such as MAPK, PI3K/AKT, and STAT signaling [46]. A second class comprises fusion proteins that act as aberrant transcription factors, such as EWSR1::FLI1 and RUNX1::RUNX1T1, leading to widespread reprogramming of gene expression networks [40, 42]. A third class includes fusions that alter epigenetic regulation and chromatin organization, as observed for SS18::SSX, BRD4::NUTM1, and JAZF1::SUZ12 [47–49]. In addition, some rearrangements activate oncogenes through enhancer hijacking or promoter swapping rather than generating classical chimeric proteins, as observed in MYC-associated rearrangements, illustrating the functional diversity of fusion-driven oncogenesis [50].

Importantly, the oncogenic effects of FTs are not limited to the encoded proteins. The FTs themselves can exhibit diverse properties, including alternative splicing, differential stability, and context-dependent expression across developmental stages or cell types, which may further modulate their biological impact. Furthermore, not all cancer-associated FTs originate from genomic rearrangements. RNA-level fusion events generated through trans-splicing, cis-splicing, read-through transcription, or circularization can also contribute to cancer development and progression and are discussed below.

## Fusion transcript prevalence across different cancer types

The prevalence of recurrent FTs varies substantially across human cancers and reflects fundamental differences in tumor biology and the mechanisms driving malignant transformation. Overall, fusion oncogenes contribute much more frequently to pediatric cancers than to adult malignancies. In a large cohort of 5,190 pediatric cancer patients, recurrent oncogenic fusions were identified in 38.8% of cases, including 55.7% of leukemias, 22.5% of brain tumors, and 18.8% of solid tumors [51]. By comparison, analysis of 4,415 adult tumors identified known oncogenic fusions in approximately 10% of cases, although the prevalence varied considerably between tumor types, ranging from 14.8% in ovarian adenocarcinoma to 5.2% in colorectal carcinoma [52].

These findings demonstrate that the contribution of FTs differs markedly according to both patient age and tissue of origin. Among the analyzed tumor types, pediatric leukemias exhibited the highest prevalence of recurrent oncogenic fusions, whereas common adult epithelial cancers, exemplified by colorectal carcinoma, exhibited among the lowest frequencies. This observation is consistent with the biology of pediatric cancers, in which recurrent fusion oncogenes often represent the primary initiating genetic event despite a relatively low overall mutational burden.

The spectrum of recurrent FTs also differs considerably between pediatric and adult cancers. In pediatric malignancies, RUNX1::RUNX1T1 and CBFB::MYH11 are among the most common recurrent fusions in leukemia, KIAA1549::BRAF predominates in pediatric brain tumors, and EWSR1::FLI1 is the characteristic driver of Ewing sarcoma [53]. Likewise, disease-defining fusions such as BCR::ABL1 in chronic myeloid leukemia, PML::RARA in APL, SS18::SSX in synovial sarcoma, and PAX3::FOXO1 in ARMS illustrate how a single recurrent genomic rearrangement can determine tumor identity, facilitate diagnosis, and, in some cases, guide therapeutic management.

By contrast, tumorigenesis in most adult epithelial cancers is driven predominantly by the progressive accumulation of point mutations, copy number alterations, and epigenetic abnormalities, whereas recurrent FTs are generally confined to molecular subsets [6]. Nevertheless, these relatively uncommon rearrangements remain clinically important because they frequently involve therapeutically targetable kinases. Recurrent kinase fusions involving ALK, RET, ROS1, NTRK, FGFR2, and FGFR3 have been identified in non-small cell lung cancer (NSCLC), thyroid carcinoma (TC), cholangiocarcinoma (CGC), glioma, pancreatic cancer (PAC), and several other epithelial malignancies, where they serve as important biomarkers for precision oncology despite their comparatively low prevalence [6, 54]. An important exception is prostate adenocarcinoma, in which the TMPRSS2::ERG fusion occurs in approximately 38% of cases, illustrating that highly recurrent fusion events can also occur in adult epithelial tumors despite their generally lower overall fusion frequencies [6].

The marked variation in FT prevalence is closely linked to tissue-specific biological mechanisms. In hematological malignancies, physiological DNA double-strand breaks generated during V(D)J recombination and immunoglobulin gene rearrangement increase susceptibility to recurrent chromosomal translocations [55]. In contrast, pediatric solid tumors generally possess relatively simple genomes with a low mutational burden, making recurrent fusion oncogenes dominant drivers of malignant transformation [56]. Adult epithelial cancers, however, evolve through the gradual accumulation of multiple genomic alterations over time and are therefore generally less dependent on individual recurrent fusion events than pediatric malignancies and hematological cancers. Collectively, these observations indicate that FTs span a biological continuum, ranging from disease-defining initiating lesions in leukemia and pediatric cancers to relatively infrequent but therapeutically actionable alterations in genetically complex adult tumors.

Table 1 summarizes representative genomic rearrangement-dependent fusion transcripts (FTs) selected to illustrate the major mechanistic and functional classes across different cancer types. The listed examples were selected based on their recurrence, biological relevance, clinical utility, and availability of functional characterization.

**Table 1:** Representative genomic rearrangement dependent FTs

Fusion	Translocation	Associated cancer types	Known function and clinical significance	Refs.
*BCR::ABL1*	t(9;22)(q34;q11)	CML, ALL	Produces a constitutively active tyrosine kinase, promotes cellular proliferation and suppresses apoptosis. Disease-defining biomarker for CML and predictive biomarker for tyrosine kinase inhibitor therapy.	[57]
*ZMYM2::FGFR1*	t(8;13)(p11;q12)	T-LBL, AML, Eosinophilia	Constitutive FGFR1 kinase activation driving myeloid and lymphoid transformation. Defines myeloid/lymphoid neoplasms with FGFR1 rearrangements and represents a potential therapeutic target for FGFR-directed therapies and targeted protein degradation strategies.	[58, 59]
*MYB::NFIB*	t(6;9)(q22-23;p23-24)	ACC	Hallmark of ACC; enhances the MYB oncogenic activity and activates MYB-regulated pathways. Useful molecular diagnostic biomarker.	[60]
*CRTC1::MAML2*	t(11;19)(q14-21;p12-13)	Mucoepidermoid carcinoma, CCC of Salivary gland	Fusion protein drives tumor growth by activating the cAMP/CREB signaling pathway. Generally associated with favorable prognosis in mucoepidermoid carcinoma.	[61]
*EWSR1::ATF1*	t(12;22)(q13;q12)	CCS	Aberrant transcription factor that upregulates c-MET and MITF expression. Characteristic diagnostic biomarker of clear cell sarcoma.	[62]
*EWSR1::FLI1*	t(11;22)(q24;q12)	Ewing Sarcoma	Fusion protein acts as an aberrant transcription factor that deregulates genes involved in tumorigenesis. Disease-defining diagnostic biomarker for Ewing sarcoma.	[42]
*EML4::ALK*	paracentric inversion inv(2)(p21;p23)	NSCLC, Lung Adenocarcinoma	The fusion protein is constitutively active and ligand-independent. It abnormally activates downstream signaling pathways, promotes cell proliferation and invasion, and inhibits apoptosis, ultimately driving NSCLC development. It can be detected by FISH and RT-PCR. Predictive biomarker for ALK-targeted therapy.	[63]
*KIF5B::RET*	inv(10)(p11.2q11.2)	Lung Adenocarcinoma, NSCLC, PTC	Constitutive RET kinase activation promotes cell proliferation through persistent downstream signaling, including STAT3 activation. Predictive biomarker for selective RET inhibitor therapy.	[64]
*KIAA1549::BRAF*	dup(7)(q34;q34)	Pilocytic Astrocytoma	Constitutive kinase activity. Diagnostic marker for PAs and potential prognostic biomarker in pediatric low-grade glioma	[65, 66]
*PCM1::JAK2*	t(8;9)(p22;p24)	MPN with eosinophilia, AML, ALL, AEL	Activates the MAPK pathway and JAK2 signaling with downstream STAT5 activation. Defines a rare subgroup of myeloid/lymphoid neoplasms that may benefit from JAK2-targeted therapies.	[67, 68]
*ETV6::NTRK3*	t(12;15)(p13;q25)	MASC, CFS, SBC, Congenital mesoblastic nephroma	Diagnostic marker for CFS. Chimeric product transforms normal mouse mammary epithelial cells. Detected by FISH and RT-PCR. Predictive biomarker for TRK inhibitor therapy.	[69, 70]
*CTNNB1::PLAG1*	t(3;8)(p21;q12)	PA, CXPA	Enhancer hijacking leading to PLAG1 overexpression. Diagnostic biomarker for pleomorphic adenoma.	[71]
*CD74::ROS1*	t(5;6)(q33.1;q22.1)	LA, NSCLC, Cholangiocarcinoma	Constitutively activates ROS1 signaling, promoting invasion and metastasis. Predictive biomarker for ROS1 inhibitor therapy.	[72]
*RUNX1::RUNX1T1*	t(8;21)(q22;q22)	AML	Dysregulates gene expression. Drives cell cycle progression in G1 by promoting CCND2 and CDK6 expression. Defines a favorable-risk AML subtype and is routinely used for diagnosis, risk stratification, and measurable residual disease (MRD) monitoring.	[73]
*PML::RARA*	t(15;17)(q24;q21)	APL	Cytogenetic hallmark of APL. Responsible for cellular transformation. Alters the nuclear structure of nuclear bodies, leading to their disruption into nuclear microspeckles in APL. Disease-defining biomarker guiding differentiation therapy.	[74]
*ETV6::RUNX1*	t(12;21)(p13.2;q22.3)	ALL	Activates EPOR transcription and alters transcriptional regulation, enhances B-cell development through RAG1 expression, promotes cell survival, and contributes to a multistep leukemogenic process. Defines a favorable-risk childhood B-ALL subtype and is routinely used for molecular classification and measurable residual disease monitoring.	[75, 76]
*PAX3::FOXO1*	t(2;13)(q35;q14)	ARMS	Drives proliferation, promotes cell survival, suppresses terminal differentiation, promotes invasion and supports angiogenesis. Used as a diagnostic aid in many pathology laboratories worldwide. Defines the ARMS subtype and is associated with unfavorable prognosis.	[43]
*NUP98::NSD1*	t(5;11)(q35.2;p15.5)	AML	Indicator of poor prognosis. Co-expression with FLT3-ITD leads to AML. Induces self-renewal and blocks differentiation of hematopoietic stem cells. High-risk AML biomarker associated with poor prognosis.	[77–79]
*‍CBFB::MYH11*	16 inv(16)(p13;q22) or t(16;16)(p13;q22)	AML	Defines the core-binding factor AML subgroup with favorable prognosis and guides risk stratification and measurable residual disease monitoring.	[80]
*‍NPM::ALK*	t(2;5)(p23;q35)	ALCL	Promotes and activates tyrosine phosphorylation of STAT3, activates the MEK/ERK pathway, PI3K, and sonic hedgehog (SHH). Upregulates several important miRNAs. Binds and activates JAK3. Disease-defining biomarker of ALK-positive ALCL and predictive biomarker for ALK-targeted therapy.	[81, 82]
*SS18::SSX*	t(x;18)(p11;q11)	Synovial sarcoma	Primary oncogenic driver of SS. Hijacks BAF (mSWI/SNF) chromatin-remodeling complexes, drives oncogenesis through disruption of the SWI/SNF complex and inappropriate activation of developmental and pro-proliferative gene programs. Diagnostic hallmark of synovial sarcoma.	[47, 83]
*TMPRSS2::ERG*	del(21)(q22)	PC	Overexpression of ERG driven by the androgen-responsive TMPRSS2 promoter, leading to transcriptional reprogramming, increased invasion, and cooperation with other oncogenic alterations in prostate cancer. Useful molecular biomarker for prostate cancer subclassification and risk assessment.	[84, 85]
*JAZF1::SUZ12*	t(7;17)(p15;q21)	ESS	Alters polycomb repressive complex 2 (PRC2) activity and epigenetic regulation; promotes aberrant transcriptional programs in endometrial stromal sarcoma. Diagnostic marker to differentiate LG-ESS and HG-ESS.	[49, 86]
*BRD4::NUTM1*	t(15;19)(q13;p13.1)	NC	Generates aberrant histone modification patterns. Creates a chromatin-binding oncoprotein that blocks differentiation through global histone acetylation. Disease-defining biomarker of NUT carcinoma	[48, 87]
*MEF2D::BCL9*	inv(1)(q21;q22)	B-ALL	Generates a transcriptional activator that enhances MEF2D-driven gene expression and promotes leukemogenesis in B-ALL. Defines a high-risk B-ALL subtype associated with poor prognosis.	[88]
*EGFR::SEPT14*	del(7)(p11.2)	Lung adenocarcinoma, Glioblastoma	Activates STAT3 signaling and confers mitogen independence and sensitivity to EGFR inhibition. Predictive biomarker for EGFR-targeted therapy.	[89]
*PTPRZ1::MET*	del(7)(q31)	Glioma	Constitutive activation of MET tyrosine kinase signaling, leading to enhanced cell proliferation, survival, and tumor growth in glioma. Potential therapeutic target through MET inhibition.	[90, 91]
*DNAJB1::PRKACA*	del(19)(p13.12)	FL-HCC	Produces a constitutively active PKA catalytic subunit, resulting in aberrant cAMP signaling, transcriptional reprogramming, and tumorigenesis in fibrolamellar hepatocellular carcinoma. Disease-defining biomarker of fibrolamellar hepatocellular carcinoma.	[92, 93]
*FUS::DDIT3*	t(12;16)(q13;p11)	Myxoid liposarcoma	Deregulates NF-κB target genes by interaction with NFKBIZ. The N-terminal FUS part is crucial for the oncoprotein’s activity. Diagnostic hallmark of myxoid liposarcoma.	[44]
*HEY1::NCOA2*	del(8)(q13.3q21.1)	Mesenchymal chondrosarcoma	Produces an aberrant transcriptional regulator that disrupts normal differentiation programs, enhances cell proliferation and drives oncogenic transcription in mesenchymal chondrosarcoma. Highly specific diagnostic biomarker of mesenchymal chondrosarcoma.	[94, 95]

Table 2 summarizes representative RNA-level fusion transcripts (FTs) generated through trans-splicing mechanisms. The listed examples illustrate this relatively uncommon class of fusion transcripts together with their underlying mechanism, associated cancer types, and reported biological functions. Compared with genomic rearrangement-derived FTs, relatively few RNA-level FTs have been functionally characterized in cancer.

**Table 2:** Representative RNA-level FTs generated independently of classical chromosomal translocations

Fusion	Mechanism / Origin	Associated cancer types	Known function	Refs.
*PAX3::FOXO1* (RNA isoform)	Trans splicing	ARMS	Transiently expressed during normal muscle differentiation; regulates MYOD/MYOG expression and promotes cell proliferation and survival.	[96]
*JAZF1::JJAZ1* (RNA isoform)	Trans splicing	ESS	Confers anti-apoptotic activity and promotes cell proliferation and survival.	[97, 98]

Table 3 summarizes representative RNA-level fusion transcripts (FTs) that arise independently of genomic rearrangements, primarily through cis-splicing and read-through mechanisms. The listed examples include their genomic location, associated cancer types, reported biological functions, and available experimental validation, thereby distinguishing functionally validated fusion transcripts from those identified primarily through sequencing-based approaches.

**Table 3:** Representative cis-splicing and read-through FTs

Fusion	Genomic location	Associated cancer types	Known function	Experimental validation	Refs.
*TFG::ADGRG7*	Chr 3 (forward strand)	T-LBL	Expressed at higher levels than the corresponding parental transcripts.	RT-PCR, Sanger sequencing	[25]
*JAK3::INSL3*	Chr 19 (reverse strand)	T-LBL	Germline read-through FT implicated in cancer predisposition.	RT-PCR, Sanger sequencing	[25]
*SLC45A3::ELK4*	Chr 1	PC	Proposed PC biomarker associated with androgen-regulated gene expression.	RT-PCR	[22]
*COMMD3::BMI1*	Chr 10	PC	Associated with higher expression of COMMD3 and has been proposed as an independent biomarker for prostate cancer	RT-PCR, qRT-PCR	[99]
*USP9Y::TTTY15*	Chr Y	PC	Frequently detected in prostate tumors, particularly in Chinese populations.	RT-PCR, sanger sequencing, FISH, qRT-PCR	[100]
*CDK12::ERBB2*	Chr 17	Breast invasive carcinoma, Gastric cancer	Internal in-frame stop codons result in the production of a truncated CDK12 protein	Sanger sequencing and RT-PCR	[101]
*‍CTAGE5::KHDRBS3*	Chr Y	PC	Function remains unclear; detected in 37% of Chinese prostate cancer patients.	RT-PCR, sanger sequencing, FISH, qRT-PCR	[100]
*SCNN1A::TNFRSF1A*	Chr 12	Breast cancer; glioblastoma	Cis-splicing of adjacent genes involved in NF-κB signaling and tumor progression.	RNA-seq, Western blot, qPCR, Sanger Sequencing	[102]
*CTSD::IFITM10*	Chr 11	Breast cancer	Promotes cell proliferation.	RNA-seq, Western blot, qPCR, Sanger Sequencing	[102]
*LHX6::NDUFA8*	Chr 9	Glioma; breast cancer; cervical cancer	May represent an early event in cervical tumorigenesis.	RT-PCR, Sanger sequencing	[103]

Table 4 summarizes representative fusion circular RNAs (f-circRNAs) identified in human cancers. The listed examples include their originating fusion genes, associated cancer types, and reported biological functions. Although only a limited number of f-circRNAs have been functionally characterized to date, growing evidence indicates that they contribute to tumorigenesis and may represent promising biomarkers and therapeutic targets.

**Table 4:** Representative fusion circular RNAs (f-circRNAs)

Fusion	Fusion gene (origin)	Associated cancer types	Known function	Refs.
f-circSR	*SLC34A2::ROS1*	NSCLC	Promotes cell migration	[104]
f-circEA1	*EML4::ALK*	NSCLC	Regulates cell proliferation and apoptosis	[105]
f-circEA-2a	*EML4::ALK*	NSCLC	Promotes cell migration and invasion	[106]
f-circEA-4a	*EML4::ALK*	NSCLC	Promotes cell migration and invasion	[106]
f-circBA9.3	*BCR::ABL1*	CML	Promotes cell proliferation and inhibits apoptosis	[107]
f-circBA1	*BCR::ABL1*	CML	Inhibits cell proliferation of CML cells by inducing cell cycle arrest.	[108]
f-circPR	*PML::RARA*	APL	Exhibits proliferative and oncogenic activity in immortalized cell models	[27, 109]
f-circEF1	*EWSR1::FLI1*	Ewing sarcoma	Enhances cell proliferation and migration. Alters the expression of genes involved in angiogenesis, immune evasion, and extracellular matrix remodeling	[27, 110]
f-circM9	*KMT2A::MLLT3*	AML	Promotes cell proliferation and transformation	[27]
f-circKA	*KANSL1::ARL17A*	Lung cancer; Medulloblastoma	Promotes cell proliferation, migration and invasion	[26]
f-circNPM-ALK	*NPM1::ALK*	ALCL, NSCLC	Acts as a miRNA inhibitor in ALCL.	[111]

## Technological advances in fusion transcript detection, characterization and functional validation

### Molecular and sequencing-based detection methods

The detection of FTs is crucial for diagnosing and understanding various cancers and genetic disorders. Several advanced molecular techniques are available for FT detection.

Fluorescence in situ hybridization (FISH) remains widely used in clinical diagnostics to visualize fusion genes directly within cells using fluorescent probes [112]. It has long been considered the gold standard for oncogenic fusion detection because of its high sensitivity and specificity and its ability to provide direct visual evidence of chromosomal rearrangements [113]. However, the resolution of FISH is limited and does not favor a high sample throughput, since it requires separate probes for each fusion to be analyzed.

**Fig. 2:**
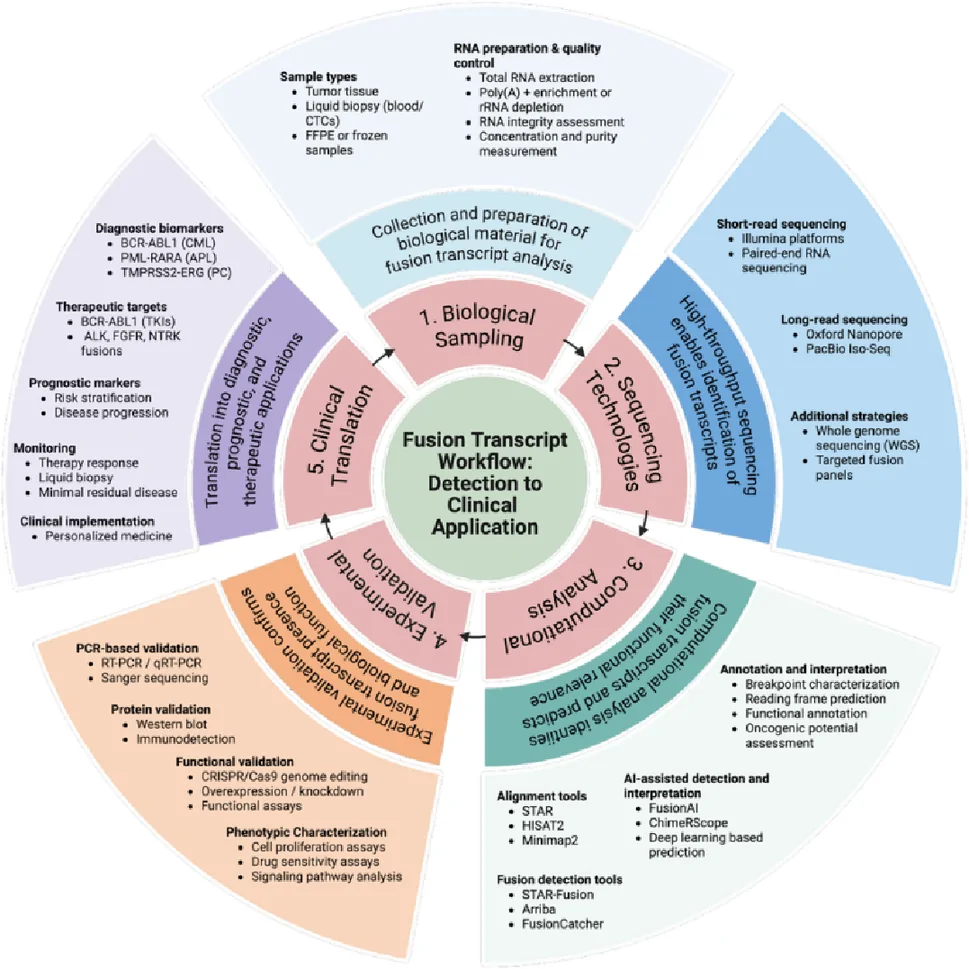
Integrated workflow for detection, validation, and clinical translation. The workflow summarizes the major stages from sample acquisition and sequencing through computational analysis, experimental validation, functional characterization, and clinical application

Detection and characterization of FTs involve a multi-step workflow comprising biological sampling, sequencing, computational analysis, experimental validation, and clinical translation (Fig. 2). The individual technologies used at each stage are discussed below.

Another prominent and long-established method is Reverse Transcription Polymerase Chain Reaction (RT-PCR), which converts RNA into complementary DNA (cDNA) and amplifies specific fusion gene sequences for easy identification (see for example Bustin and Mueller 2005 [114], Kaltenboeck and Wang 2005 [115]). In addition, targeted sequencing approaches such as hybrid capture-based panels and anchored multiplex PCR (AMP) have become widely used in clinical diagnostics, enabling sensitive detection of known and novel FTs with reduced cost and turnaround time compared to whole genome sequencing [116, 117]. Additionally, techniques such as microarray-based assays (e.g. Skotheim et al. 2009 [118]) and digital droplet PCR (ddPCR; compare Coccaro et al.2020 [119]) offer precise quantification and detection of FTs, thus contributing to the enhancement of diagnostic accuracy.

A more recent and powerful approach is next-generation sequencing (NGS), particularly RNA sequencing (RNA-seq), which provides comprehensive information on the transcriptome. Long-read sequencing technologies, such as Oxford Nanopore and PacBio platforms, further enhance FT detection by enabling full-length transcript sequencing and more accurate identification of complex fusion structures and isoforms. These approaches enable the detection of both known and novel FTs. Individually or in combination, these methods enable robust detection and characterization of FTs. However, NGS-based approaches are prone to false positive fusion calls arising from sequencing artefacts, alignment errors, or read-through transcription events, necessitating careful computational filtering and experimental validation.

NGS-based techniques in particular open up new avenues for the analysis and detection of cancer-related alterations (recently reviewed for example by Mackinnon et al. 2024 [120], Stenzinger et al. 2024 [121]).

Whole genome sequencing (WGS), for example is a powerful tool in cancer genetics, since it offers comprehensive insights into the entire genetic landscape of a tumor at the DNA-level with single nucleotide resolution. WGS provides an unbiased approach for detecting structural variants and gene fusions across the entire genome, enabling the discovery of rare and previously unknown fusion events. In addition, WGS can identify co-occurring mutations and structural alterations that may contribute to disease progression or therapy resistance. One challenge is the vast amount of data generated by WGS, which requires expertise and sophisticated bioinformatics tools in order to be able to correctly identify gene fusions [122]. Additional limitations include cost and turnaround time. While costs have dropped drastically, WGS still remains rather expensive compared to targeted approaches. Furthermore, data analysis and interpretation can be time-consuming, which may be disadvantageous in time-sensitive clinical settings.

A promising but still costly NGS-based approach is single-cell sequencing, which allows analysis of genetic material at the level of individual cells. Among single-cell approaches, single-cell RNA sequencing (scRNA-seq) has emerged as a particularly valuable tool for FT detection and characterization. The scRNA-seq technique involves the isolation of individual cancer cell populations, reverse transcribing their RNA into cDNA, and then sequencing the cDNA to identify and quantify transcripts (see e.g. Iacobucci et al. 2023 [123], Paas-Oliveros et al. 2023 [124], Patton and Dermawan 2024 [125]). Single-cell sequencing offers several advantages for FT analysis. It enables the detection of rare fusion events present only in small cellular subpopulations, provides insights into intratumoral heterogeneity and clonal evolution, and can reveal FTs associated with therapy resistance or disease relapse. Recent integration with spatial transcriptomics further allows the localization of FT-expressing cells within the tumor microenvironment. Taken together, single-cell sequencing provides a powerful approach for investigating FT heterogeneity at cellular resolution. Despite these advantages, the detection of FTs in single-cell data remains challenging due to low sequencing depth and technical dropout, which can limit sensitivity.

The large number of candidate FTs generated by sequencing-based approaches requires specialized computational pipelines for fusion calling, artefact filtering, annotation, and biological prioritization. These computational strategies, including conventional alignment-based algorithms and emerging artificial intelligence-assisted methods, are discussed in the following section.

### Computational and AI-assisted approaches for fusion transcript detection

Building on the experimental and sequencing-based detection strategies described above, recent advances in computational methods, particularly artificial intelligence and machine learning, have significantly improved the identification and interpretation of FTs. Over the past decade, artificial intelligence (AI), machine learning and deep learning, together with other bioinformatic approaches, have transformed how complex biological data are analyzed. Modern sequencing technologies generate massive amounts of genomic and transcriptomic data, which often contain subtle patterns that are difficult to capture using traditional rule-based algorithms. Deep learning models provide a powerful solution to this challenge, as they can automatically learn patterns from raw data [126–128]. In cancer research, these approaches are being applied to somatic variation detection, structural variation detection, mutation classification, and more recently, FT identification and breakpoint prediction [129–131]. Prior to the integration of AI-driven strategies, fusion detection primarily relied on conventional NGS-based methods. These approaches identify candidate fusions through the analysis of split reads and discordant read pairs that map to different genes. While these methods have proven to be effective, they are susceptible to false-positive results, particularly in repetitive regions or in lowly expressed genes. Modern pipelines are incorporating data-driven filtering strategies and machine learning models trained on validated fusion datasets to address these limitations. Tools such as STAR-Fusion [132] and Arriba [133] exemplify this evolution. Both methods rely on high-quality read alignment to detect candidate fusion junctions and apply refined filtering strategies to prioritize biologically meaningful events. It has been demonstrated that both of these tools offer several additional features, including the prediction of the protein coding status of the fusion. Although based on a similar workflow, Arriba has demonstrated high precision and sensitivity in comparative benchmarking studies. It does not require the modification of STAR alignment parameters and avoids downstream processing impairments [133]. Arriba has been successfully implemented in routine whole-transcriptome diagnostics. In a large cohort of 1233 FFPE solid tumors analyzed at the Institute of Pathology at Heidelberg University, approximately 92% sensitivity for known fusion-positive cases was achieved. The false negative cases were mainly associated with low tumor cell content or atypical event types [134]. STAR-Fusion has also been used at scale in a CLIA-certified setting, analyzing 4800 IDH-wildtype glioblastomas. It achieved ≥ 97% positive and ≥ 99% overall agreement with validated comparator methods. Recurrent fusions such as FGFR3::TACC3 and PTPRZ1::MET were identified for clinical reporting [135]. Together, these studies illustrate that both tools are now embedded in routine RNA-seq diagnostics. This includes the identification of targetable kinase fusions, for example, EML4::ALK and KIF5B::RET in non-small cell lung cancer [136, 137].

Beyond improving alignment-based detection, deeper learning architectures are driving fusion research towards predictive modeling. FusionAI represents a different conceptual advance by operating at the DNA sequence level rather than exclusively at the transcript level [130]. Using convolutional neural networks trained on thousands of curated breakpoint sequences from large fusion databases, FusionAI learns patterns within extended genomic regions surrounding breakpoints. By analyzing flanking DNA context, FusionAI predicts genomic regions that may be predisposed to fusion formation. This approach provides an additional layer of validation for RNA-based fusion calls and helps distinguish true structural rearrangements from artefacts generated during sequencing or alignment.

Recent deep-learning variant callers such as DeepSomatic demonstrate the capacity of neural-network models to analyze heterogeneous sequencing platforms and challenging clinical specimens, including FFPE samples. While these models were initially developed for detection of small nucleotide variants, they exemplify the broader transition toward data-driven models capable of interpreting increasingly complex genomic alterations. This conceptual advance is currently being applied to the fields of FT detection and breakpoint analysis.

Long-read sequencing technologies have significantly expanded the possibilities for fusion analysis. Short-read RNA-seq can fragment complex fusion junctions, but long-read platforms such as PacBio and Oxford Nanopore can capture entire FTs in single reads [138]. Computational frameworks like CTAT-LR-Fusion use this advantage to map complex junction architectures with higher resolution, resolve multiple isoforms of the same fusion, and clarify breakpoint structures that may be ambiguous in short-read data [139]. This higher structural resolution is vital for tumors with complex genomic rearrangements.

Other deep learning-based approaches avoid alignment-related challenges. Alignment-free strategies, such as those implemented in ChimeRScope, use a k-mer based fingerprinting approach to rapidly identify candidate FTs [140]. This approach breaks RNA-seq reads into small fragments and matches them against a pre-built reference library, thereby pinpointing sequences that bridge two distinct genes. These models can mitigate mapping errors in repetitive or low-complexity genomic regions by reducing dependence on reference genome alignment. Meanwhile, integrative frameworks like FusionPro adopt an ensemble strategy, bridging both genomics and proteomics data [141]. Combining information from RNA sequencing and mass spectrometry allows the detection of FTs and the actual fusion-derived proteins produced in cells. This integrated approach makes it easier to study fusion junction isoforms and build sample specific, customized databases for further MS/MS analysis. This approach facilitates the identification of new biomarkers or therapeutic targets.

A large benchmarking study by Haas et al. evaluated 23 fusion detection tools using both simulated and real cancer RNA-seq data. Read-mapping approaches, particularly STAR-Fusion and Arriba, emerged as the most accurate and fastest methods, supporting their use in research and clinical settings. This study highlights that algorithm performance depends on the characteristics of the sequencing dataset and the intended application, providing an important framework for selecting appropriate fusion detection methods [142].

Together, these computational approaches illustrate the rapid evolution of FT detection, from conventional read-mapping algorithms to AI-assisted prediction and multi-omics integration. Their principal characteristics, applications, strengths, and limitations are summarized in Table 5.

**Table 5:** Representative computational tools for fusion transcript detection, characterization, and prioritization

Platform / Workflow	Tool	Detection strategy	Primary application	Key strengths	Limitations	Ref.
Short-read RNA-seq	STAR-Fusion	STAR alignment with split-read and discordant-pair detection	General fusion discovery	High accuracy; fast; widely benchmarked	Sensitive to sequencing depth and expression; limited IGH benchmarking	[142]
Short-read RNA-seq	Arriba	STAR-based fusion calling with extensive artefact filtering	Clinical and research RNA-seq	High sensitivity and precision; rapid runtime; clinically implemented	Performance depends on RNA quality, tumor purity, and filtering settings	[133, 142]
Short-read RNA-seq	FusionCatcher	Multi-aligner and multi-database integration	Broad discovery of known and novel fusions	Comprehensive fusion detection	High computational demand; relatively large candidate sets	[143]
Short-read RNA-seq	CICERO	Local assembly of clipped and junction reads	Complex and cryptic rearrangements	Excellent for non-canonical driver alterations	Requires sufficient junction coverage; optimized for driver events	[144]
Short-read RNA-seq	MetaFusion	Consensus integration of multiple callers	High-confidence fusion prioritization	Improved precision and recall over individual callers	Requires outputs from multiple fusion callers	[145]
Long-read RNA-seq	JAFFAL	Long-read alignment-based fusion detection	Full-length fusion and isoform analysis	Resolves complete transcript structures	Influenced by long-read error rates and sequencing depth	[146]
Long-read RNA-seq	CTAT-LR-Fusion	Long-read fusion calling and isoform reconstruction	Complex fusion architecture and isoform analysis	High structural resolution; supports bulk and single-cell data	Requires adequate long-read coverage	[139]
Whole-genome sequencing	Manta	Structural variant detection from DNA sequencing	Genomic breakpoint identification	Sensitive DNA structural variant detection	RNA validation required to confirm expressed FTs	[147]
Proteogenomics	FusionPro	RNA-seq with optional MS validation	Fusion protein prediction and validation	Integrates transcriptomic and proteomic evidence	Protein validation depends on MS coverage; not a primary fusion caller	[141]
AI / Deep learning	FusionAI	CNN-based breakpoint prediction	Candidate breakpoint prioritization	Predicts genomic regions susceptible to fusion formation	Does not directly detect expressed fusion transcripts	[130]

Note: Performance metrics reported in different studies are not directly comparable because benchmarking datasets, sequencing platforms, library preparation, filtering criteria, and definitions of true-positive fusion events vary substantially. IGH-associated fusions remain particularly challenging because of repetitive genomic regions, physiological V(D)J recombination, and variable breakpoint locations. Consequently, tool performance should be interpreted within the context of the original benchmarking studies

Recent methodological advances have shifted fusion detection from conventional alignment-based algorithms toward integrated computational frameworks combining long-read sequencing, machine learning, ensemble calling strategies, and orthogonal experimental validation. Although these computational approaches differ substantially in methodology and intended application, several common principles emerge from comparative benchmarking studies.

Overall, comparative benchmarking indicates that no single computational algorithm is optimal for every experimental setting. Read-mapping approaches such as STAR-Fusion and Arriba currently provide the best balance between sensitivity, precision, and computational efficiency for short-read RNA sequencing, whereas long-read methods excel at resolving complex fusion structures and transcript isoforms. Machine learning and artificial intelligence-based approaches are increasingly complementing conventional pipelines by improving candidate prioritization, reducing false-positive calls, and predicting biologically relevant breakpoint regions. Future computational workflows will likely integrate multiple complementary algorithms rather than relying on a single detection strategy [132, 142].

Detection performance may also vary depending on fusion architecture and genomic context. Immunoglobulin (IGH)-associated rearrangements in hematological malignancies represent a particularly challenging category because physiological V(D)J recombination, extensive sequence diversity, and repetitive genomic regions complicate accurate breakpoint detection. Long-read sequencing approaches can improve the resolution of complex IGH rearrangements by spanning repetitive regions and resolving complete fusion structures, while single-cell sequencing approaches may facilitate detection of rare fusion-positive subpopulations masked in bulk sequencing analyses. However, these approaches remain limited by higher costs, computational complexity, and the need for further optimization of analytical workflows [148]. Accurate computational detection represents only the first step toward identifying biologically and clinically relevant fusion events. Despite these advances, computational detection alone does not establish the biological or clinical relevance of a FT. Many sequencing studies identify large numbers of candidate fusion events, of which a substantial proportion may represent passenger alterations or technical artefacts. Therefore, computational approaches should be considered a prioritization step rather than definitive proof of functional relevance. Current prioritization strategies integrate multiple biological features, including recurrence across patient cohorts, expression level, preservation of open reading frames, predicted protein domains, breakpoint characteristics, evolutionary conservation, and pathway involvement, to identify candidate driver fusions for experimental investigation. Consequently, computational predictions should be complemented by experimental validation. Following computational prioritization, functional validation is essential to distinguish oncogenic driver fusions from incidental events. Scalable experimental approaches, including CRISPR/Cas9-based genome engineering, CRISPR interference or activation screens, organoid models, and patient-derived xenografts (PDX), provide complementary strategies to evaluate the transforming potential, cellular dependency, and therapeutic vulnerability of candidate FTs. Integration of AI-based prioritization with high-throughput functional validation approaches may substantially accelerate the discovery of clinically relevant fusion drivers while reducing the experimental burden associated with evaluating thousands of newly identified fusion events [149, 150].

Although artificial intelligence has substantially improved fusion detection and breakpoint prediction, its application remains constrained by the quality and diversity of available training datasets, limited model interpretability, and the lack of standardized benchmarking across different sequencing platforms [151]. Future advances in explainable AI, standardized evaluation frameworks, and integrated computational–experimental pipelines are expected to improve the biological interpretation and clinical translation of FT predictions. Nevertheless, orthogonal validation using RT-PCR, Sanger sequencing, fluorescence in situ hybridization (FISH), or long-read sequencing remains indispensable, particularly for low-abundance or clinically actionable FTs.

### CRISPR/Cas9-based generation and functional modeling of fusion transcripts

CRISPR/Cas9 technology has transformed the generation of experimental FT models by enabling precise engineering of chromosomal rearrangements, gene fusions, and fusion-associated genomic alterations [152]. The system enables targeted genome editing in both in vitro and in vivo models through the programmable introduction of DNA double-strand breaks at selected genomic loci. CRISPR/Cas9 can be used to recreate cancer-associated chromosomal rearrangements, including translocations, inversions, deletions, and tandem duplications, which subsequently generate FTs under endogenous regulatory control. In vitro, CRISPR/Cas9 is commonly used to engineer FT models in established cell lines or primary cells. In vivo, the CRISPR/Cas9 approach allows targeting specific tissues or organs to induce the formation of FTs there. Thus, this technique offers a flexible and efficient method for modeling chromosomal rearrangements. It serves as an alternative to traditional methods for the generation of transgenic or knock-in models in various biological and cancer research studies. The general principles, delivery strategies, and therapeutic applications of CRISPR/Cas9 have been reviewed extensively by Li et al. [149]. Briefly, CRISPR/Cas9 vectors together with guide RNAs are designed to target specific genomic loci and induce double-strand breaks, which are subsequently repaired by endogenous DNA repair pathways, ultimately resulting in the desired fusion event.

Using CRISPR/Cas9-based strategy, chromosomal translocations characteristic of hematologic malignancies can also be engineered by introducing paired double-strand breaks at endogenous gene loci. For example, the BCR::ABL1 fusion, which defines the Philadelphia chromosome-positive leukemias, can be generated by targeting the BCR and ABL1 loci simultaneously, resulting in precise fusion formation within the native genomic context. This approach enables direct modeling of leukemia-associated translocations and provides a controlled system to study fusion-driven signaling and cellular phenotypes [153].

A well-known example is EML4::ALK, which arises through a paracentric inversion on chromosome 2 and generates a constitutively active ALK kinase. Blasco et al. demonstrated that paired CRISPR/Cas9-mediated cleavage at the endogenous Eml4 and Alk loci efficiently generated the rearrangement both in cultured cells and directly in mouse lungs in vivo, leading to rapid tumor formation [154].

Beyond conventional cell lines and animal models, CRISPR/Cas9-generated FTs can also be studied in induced pluripotent stem cells (iPSCs). These systems provide opportunities to investigate developmental-stage-specific effects of oncogenic fusions and enable patient-specific disease modeling. Examples include KMT2A::AFF1-driven hematopoietic reprogramming and SERPINE1::FOSB models of pseudomyogenic hemangioendothelioma [155–158]. However, iPSC-based systems remain technically demanding and may be affected by genomic instability, incomplete differentiation, and variable maturation states, which can influence the faithful modeling of disease phenotypes [159].

In addition, genome editing can be combined with advanced model systems such as patient-derived organoids and PDXs to investigate fusion-driven tumor biology in more physiologically relevant settings. Patient-derived organoids and PDXs models provide additional platforms for investigating fusion-driven tumor biology in more physiologically relevant settings. When combined with genome editing, these models can be used to introduce, correct, or functionally validate candidate fusion events while preserving aspects of tissue architecture or patient-specific tumor biology. Nevertheless, organoid and PDX systems remain labor-intensive, are not readily scalable for large functional screens, and cannot fully recapitulate the genetic, microenvironmental, and evolutionary complexity of human tumors [150, 160].

Despite CRISPR/Cas9 being the predominant platform for experimental modeling of fusion-associated genomic rearrangements, several alternative genome-engineering and gene-delivery strategies have also been employed to generate fusion-associated models. Before the introduction of CRISPR/Cas9, zinc-finger nucleases (ZFNs) and transcription activator-like effector nucleases (TALENs) were widely used to generate targeted DNA double-strand breaks and could produce defined chromosomal rearrangements with high specificity. However, both approaches require the engineering of sequence-specific DNA-binding proteins for each target locus, making experimental design more labor-intensive and less flexible than guide RNA-based CRISPR/Cas9 systems. Compared with ZFNs, TALENs generally offer simpler target design and reduced off-target activity, but both technologies remain less suitable for multiplex genome editing and high-throughput applications. In addition, adeno-associated virus (AAV)-mediated knock-in strategies enable the precise introduction of predefined fusion constructs through homology-directed repair and remain valuable for generating selected isogenic cellular and animal models under endogenous regulatory control. However, these approaches are constrained by limited vector cargo capacity and relatively low knock-in efficiency. Overall, the simplicity of guide RNA design, efficient multiplex genome editing, and greater experimental versatility have established CRISPR/Cas9 as the preferred platform for modeling cancer-associated FTs, although ZFNs, TALENs, and AAV-mediated approaches continue to provide valuable alternatives in specific experimental settings [161, 162].

Despite these advantages, CRISPR/Cas9-based fusion generation is subject to several important technical limitations. Off-target cleavage, variable DNA repair outcomes, low frequencies of the intended chromosomal rearrangement, and unintended genomic alterations may influence both the efficiency and reproducibility of engineered models [162]. Moreover, experimentally generated FTs may not fully reproduce the epigenetic, developmental, or clonal context in which the corresponding rearrangements arise naturally during tumor evolution. Consequently, phenotypic findings and therapeutic responses observed in engineered models may not always fully predict treatment responses in patients and should therefore be interpreted together with complementary patient-derived models and clinical evidence. Careful molecular validation of both the genomic breakpoint and the expressed FT is therefore essential before downstream functional analyses are performed. Nevertheless, CRISPR/Cas9-based models provide a powerful experimental platform for linking FT discovery to mechanistic investigation, enabling direct assessment of oncogenic function, cellular phenotypes, and potential therapeutic vulnerabilities.

## Clinical applications

One of the most important clinical applications of FTs is their use as diagnostic, prognostic, and predictive biomarkers. In several malignancies, recurrent FTs define distinct disease entities, contribute to risk stratification, guide treatment selection, and enable molecular monitoring of therapeutic response. Representative examples across hematological malignancies and solid tumors are discussed below, while their broader biological and clinical significance is summarized in Table 1.

The presence of the BCR::ABL1 FT in CML is one of the best-established examples of a clinically relevant fusion biomarker and is routinely used for diagnosis, disease monitoring, and assessment of treatment response. In AML, several recurrent gene fusions and rearrangements have important diagnostic and prognostic relevance, including RUNX1::RUNX1T1, PML::RARA, DEK::NUP214, NUP98::NSD1, CBFB::MYH11, CBFA2T3::GLIS2, RBM15::MKL1, KAT6A::CREBBP, and various KMT2A rearrangements. Several of these alterations are incorporated into contemporary molecular classifications of AML [163]. In particular, RUNX1::RUNX1T1 and CBFB::MYH11 characterize core-binding-factor AML, which is generally associated with favorable prognosis and is routinely monitored during treatment by molecular methods. Furthermore, FT status is incorporated into prognostic scoring systems and contributes to therapeutic decision-making, illustrating the central role of FT profiling in modern AML management [164, 165].

FTs also serve as important diagnostic and predictive biomarkers in solid tumors. EML4::ALK is an established predictive biomarker for ALK inhibitor therapy in NSCLC [166], whereas TMPRSS2::ERG is widely used as a diagnostic biomarker in PC [167].

Human secretory breast carcinoma is characterized by the ETV6::NTRK3 fusion in approximately 92% of cases, making this alteration a highly valuable diagnostic marker [69]. Similarly, the DNAJB1::PRKACA fusion is highly specific for fibrolamellar hepatocellular carcinoma and functions as a major oncogenic driver, making it an attractive therapeutic target [168]. The CD44::SLC1A2 FT has been identified specifically in gastric adenocarcinoma and primary colorectal carcinoma, but not in corresponding normal tissues, supporting its potential diagnostic utility. Functional studies further suggest that CD44::SLC1A2 promotes cellular proliferation, invasion, and anchorage-independent growth in gastric adenocarcinoma [169, 170].

Although advances in sequencing technologies and computational algorithms have substantially improved FT detection, accurate clinical interpretation remains dependent on functional validation, molecular context, and disease-specific biological characteristics.

The clinical relevance of FTs extends beyond diagnosis and risk stratification to precision oncology. For example, more than 50 recurrent fusion genes have been reported in TC, the majority involving receptor tyrosine kinase genes. These alterations represent clinically actionable therapeutic targets, and several fusion-directed therapies, including pralsetinib, selpercatinib, and larotrectinib, are now incorporated into the clinical management of selected patients with advanced TC [171].

Despite their important diagnostic and therapeutic value, FTs rarely determine clinical outcome in isolation. Their prognostic significance is frequently modified by cooperating genomic alterations. A representative example is B-ALL, where additional genomic lesions modify risk stratification and treatment outcome. In particular, deletion of IKZF1 has been associated with adverse prognosis in B-cell precursor ALL, with particularly strong clinical relevance in BCR::ABL1-positive disease, suggesting that loss of B-cell developmental regulators contributes to treatment resistance and relapse risk [172]. More broadly, the IKZF1plus genomic profile, characterized by IKZF1 deletion together with additional deletions affecting genes involved in tumor suppression and B-cell development, defines a high-risk subgroup of B-cell precursor ALL with inferior outcomes [173]. These findings demonstrate that the clinical significance of a FT cannot be interpreted independently of the broader genomic context and that comprehensive molecular profiling is required for accurate risk assessment and treatment stratification.

A unique feature of several pediatric leukemias is that oncogenic FTs can arise long before clinical disease becomes apparent. Increasing evidence indicates that several recurrent pediatric leukemia-associated FTs originate during fetal development. For example, ETV6::RUNX1, one of the most common FTs in childhood B-cell acute lymphoblastic leukemia (B-ALL), has been detected in neonatal blood spots and umbilical cord blood at a frequency of approximately 5% of healthy newborns, demonstrating that the initiating chromosomal translocation frequently occurs in utero [174]. However, the incidence of childhood B-ALL is substantially lower, indicating that most prenatal pre-leukemic clones never progress to overt leukemia. These observations suggest that ETV6::RUNX1 alone is insufficient for leukemogenesis and that additional postnatal genetic alterations are required for malignant transformation [175]. Similar evidence has recently been reported for ZNF384 fusion-positive leukemia in monozygotic twins, in whom a shared prenatal ZNF384 fusion was followed by the independent acquisition of secondary mutations, including PTPN11 alterations, ultimately resulting in leukemia in both children [176]. Collectively, these observations support a multistep model of pediatric leukemogenesis in which FTs establish clinically silent pre-leukemic clones during fetal development, whereas subsequent cooperating genetic alterations and selective pressures drive progression to overt leukemia during childhood.

Despite remarkable advances in FT detection, biological interpretation remains a major challenge because only a small fraction of detected FTs has been experimentally validated. Consequently, distinguishing functional driver fusions from biologically neutral passenger events remains an important obstacle for clinical translation. Nevertheless, a subset of well-characterized oncogenic fusions has progressed beyond biomarker use and now represents direct therapeutic vulnerabilities.

## Fusion transcripts as therapeutic targets

The identification of oncogenic fusion transcripts has transformed precision oncology and provided some of the most successful examples of biomarker-guided targeted therapy. However, the therapeutic relevance of a fusion event depends on the biological function of the resulting fusion protein. Fusion proteins containing constitutively active enzymatic domains, particularly tyrosine kinases, have been the most amenable to pharmacological intervention, whereas transcription factor and chromatin-associated fusions remain more challenging targets.

The BCR::ABL1 fusion in CML represents the paradigm for fusion-directed therapy. The constitutively active ABL1 kinase generated by BCR::ABL1 can be effectively inhibited by ATP-competitive TKIs, including first-, second-, third-generation TKIs, as well as the allosteric ABL1 inhibitor asciminib [153, 177–179]. Despite these advances, acquired resistance remains a major clinical challenge. Resistance mechanisms include amplification of the BCR::ABL1 fusion, activation of alternative signaling pathways, and, most importantly, mutations within the ABL1 kinase domain. The T315I gatekeeper mutation is particularly relevant because it confers resistance to many first- and second-generation TKIs, although it can be effectively targeted by ponatinib and asciminib-based strategies [180].

The success of BCR::ABL1-targeted therapy has established a general therapeutic paradigm that extends to several other oncogenic kinase fusions. In non-small cell lung cancer (NSCLC), EML4::ALK defines a molecular subgroup that benefits from ALK inhibition. Although crizotinib established the feasibility of targeting ALK fusions, next-generation inhibitors such as alectinib, brigatinib, and lorlatinib have improved systemic and central nervous system activity [181]. However, resistance can develop through secondary ALK kinase domain alterations, including solvent-front and gatekeeper mutations, highlighting the need for sequential treatment strategies and development of inhibitors with activity against resistant variants [182]. Likewise, RET and NTRK fusions have become important therapeutic targets. Selective RET inhibitors such as selpercatinib and pralsetinib and TRK inhibitors such as larotrectinib and entrectinib demonstrate that fusion-driven cancers can be effectively treated across different tumor types when the molecular alteration is accurately identified [183, 184].

Fusion transcripts are not always represented by a single molecular entity. Alternative splicing, different breakpoint locations, and promoter usage can generate multiple fusion isoforms with distinct coding potential, protein domains, and biological activities. This isoform diversity has important implications for both diagnosis and treatment. For example, different BCR::ABL1 transcript variants (such as e13a2 and e14a2) produce proteins with slightly different molecular characteristics and are routinely considered in molecular diagnostics and monitoring strategies. Similarly, alternative isoforms of kinase fusions may influence the presence of functional domains required for inhibitor binding and may contribute to differences in therapeutic response [185]. Therefore, accurate characterization of fusion isoforms is essential for selecting appropriate targeted therapies and designing reliable diagnostic assays. Long-read sequencing approaches, which can capture full-length fusion transcripts, provide an important advantage for resolving isoform complexity compared with conventional short-read sequencing approaches [186].

Emerging approaches such as targeted protein degradation may further expand the therapeutic potential of fusion-driven cancers. Proteolysis-targeting chimeras (PROTACs) induce selective degradation of target proteins by recruiting the ubiquitin–proteasome system and may overcome limitations of conventional inhibitors, particularly for fusion proteins lacking enzymatically active domains. This approach is especially attractive for difficult-to-target oncogenic fusions involving transcription factors or chromatin regulators, such as SS18::SSX and BRD4::NUTM1. Although most fusion-directed PROTAC strategies remain in preclinical development, advances in degrader design, delivery, and E3 ligase recruitment may provide new opportunities to target previously inaccessible fusion proteins [187, 188].

Additional therapeutic strategies are being explored to selectively suppress fusion transcript expression, including antisense oligonucleotides, RNA interference, and CRISPR-based RNA-targeting approaches [189, 190]. Although these technologies remain largely preclinical, they offer the possibility of directly targeting fusion transcripts that encode otherwise undruggable proteins.

Beyond direct pharmacological inhibition or degradation of fusion proteins, fusion-specific sequences may also create opportunities for tumor-directed immunotherapy. Fusion-derived neoantigens represent an attractive target for personalized immunotherapy because the fusion junction creates novel peptide sequences that are absent from normal tissues. These junction-derived peptides can be processed and presented by human leukocyte antigen (HLA) molecules and recognized by T cells, providing highly tumor-specific targets. For example, BCR::ABL1-derived peptides have been shown to elicit T-cell responses, while the EWSR1::WT1 fusion in desmoplastic small round cell tumor has been directly identified as an HLA-presented neoantigen, supporting the feasibility of fusion-targeted immunotherapy [191]. Computational approaches integrating fusion detection with HLA typing and peptide-MHC binding prediction are increasingly used to prioritize candidate neoepitopes for experimental validation. However, clinical translation remains challenging because many fusion transcripts are expressed at low levels, exhibit limited immunogenicity, or occur only in subclonal tumor populations. Consistent with observations from the broader neoantigen field, subclonal neoantigens are associated with reduced tumor-reactive T-cell responses and less favorable outcomes following immune checkpoint blockade. These findings suggest that clonally conserved fusion-derived neoantigens may represent more effective immunotherapeutic targets than subclonal fusion events [192].

Combination therapies are increasingly recognized as essential because inhibition of a single oncogenic fusion is often insufficient to achieve durable disease control. Co-existing genomic alterations, pathway activation, and adaptive cellular responses can reduce therapeutic effectiveness. Consequently, future therapeutic strategies will increasingly integrate fusion-directed therapies with comprehensive molecular profiling, immunotherapy, rational combination approaches, and emerging RNA-targeting technologies to improve patient stratification, overcome resistance mechanisms, and achieve more durable clinical responses [53].

## Conclusions and future perspectives

FTs have emerged as important molecular features in cancer biology, serving as biomarkers, therapeutic targets, and mechanistic drivers across diverse malignancies. Beyond their established role as biomarkers, FTs represent a biologically diverse class of molecular events, ranging from potent oncogenic drivers to passenger alterations with limited functional relevance. Distinguishing clinically actionable fusions from functionally neutral events therefore remains a central challenge in the field.

Although thousands of cancer-associated FTs have been identified, only a small fraction is currently used in clinical practice. Many remain poorly characterized due to limited functional validation, incomplete annotation, and challenges in detection. In particular, lowly expressed FTs and complex transcript isoforms remain challenging to detect and characterize. Importantly, not all FTs are directly associated with cancer. Some are detectable in healthy tissues without any apparent association with disease, whereas others arise from genes that are not classical oncogenes.

Recent technological advances are beginning to address these limitations. Long-read sequencing enables improved resolution of FT structure by capturing full-length transcripts, while single-cell and spatial transcriptomic technologies together with integrative multi-omics approaches are providing unprecedented opportunities to better understand the biological and clinical consequences of fusion transcripts. However, despite these developments, the biological and clinical relevance of most FTs remains unclear.

In parallel, functional model systems, including CRISPR-engineered cell lines, animal models, and induced pluripotent stem cell (iPSC)-based approaches, provide important platforms to investigate fusion-driven mechanisms in a controlled setting. These model systems enable the study of specific fusion events and their downstream consequences and support functional validation, drug discovery, resistance research, and immunotherapy development. Each model system has inherent limitations, including incomplete representation of tumor heterogeneity and microenvironmental influences. In the case of iPSC-based models, additional challenges include genomic instability, incomplete differentiation, and variable maturation states. Nevertheless, patient-derived and patient-specific models, including iPSC-based systems, offer unique opportunities to investigate FT biology in a personalized context and may facilitate the development of tailored therapeutic strategies.

Emerging approaches such as liquid biopsy-based detection of circulating FTs also offer promising opportunities for non-invasive diagnostics and disease monitoring, further expanding their clinical applicability.

Taken together, the major challenge in the field is no longer the identification of FTs, but their interpretation and prioritization. While technological advances have enabled the detection of an ever-growing number of FTs, only a minority represent true drivers of tumorigenesis, whereas many may reflect context-dependent or biologically neutral processes. Bridging this gap will require integrative strategies combining high-resolution sequencing, artificial intelligence-assisted computational analysis, multi-omics integration, and systematic functional validation.

Despite these remarkable advances, several key questions remain to be addressed. How can clinically relevant FTs be distinguished from passenger events in a scalable manner? How can functional validation keep pace with the rapid rate of discovery? How can artificial intelligence, multi-omics integration, and single-cell technologies improve the biological interpretation of fusion transcripts across diverse cellular contexts? How can emerging therapeutic strategies, including targeted protein degradation, RNA-targeting technologies, and immunotherapy, be translated into durable clinical benefit?

Ultimately, addressing these challenges will be essential to move the field beyond descriptive cataloging toward a predictive, mechanistically informed, and clinically actionable understanding of fusion transcripts in cancer. Continued integration of advanced sequencing technologies, artificial intelligence, functional genomics, and precision medicine is expected to accelerate this transition and further establish fusion transcripts as central pillars of precision oncology.

## Data Availability

No datasets were generated or analysed during the current study.

## Abbreviations

AAV: Adeno-associated virus
ACC: Adenoid cystic carcinoma
AEL: acute erythroid leukemia
AFH: Angiomatoid fibrous histiocytoma
AI: Artificial intelligence
ALCL: Anaplastic large cell lymphoma
ALK: Anaplastic lymphoma kinase
ALL: Acute lymphoblastic leukemia
AMbL: Acute megakaryoblastic leukemia
AMKL: Acute megakaryocytic leukemia
AML: Acute myeloid leukemia
AMP: Anchored multiplex PCR
APL: Acute Promyelocytic Leukemia
ARMS: Alveolar rhabdomyosarcoma
ATO: Arsenic trioxide
ATRA: All-trans retinoic acid
B-ALL: Precursor B-cell acute lymphoblastic leukemia
BC: Breast Cancer
BL: Burkitt Lymphoma
CC: Colon Cancer
CCS: Clear cell Sarcoma
CCSLGT: Clear cell sarcoma-like tumor of the gastrointestinal tract
CFS: Congenital fibrosarcoma
CGC: Cholangiocarcinoma
CML: Chronic myeloid leukemia
CRC: Colorectal cancer
CRISPR: Clustered regularly interspaced short palindromic repeats
CXPA: Carcinoma ex-pleomorphic adenoma
ddPCR: Digital droplet PCR
DLBCL: Diffuse large B-cell lymphomas
EA: Esophageal adenocarcinoma
EFS: Event-free survival
ERMS: Embryonal rhabdomyosarcoma
ESS: Endometrial stromal sarcoma
ETP-ALL: Early T-cell precursor acute lymphoblastic leukemia
f-circRNA: Fusion circular RNA
FISH: Fluorescence in situ hybridization
FL-HCC: Fibrolamellar hepatocellular carcinoma
FT: Fusion transcript
GA: Gastric adenocarcinoma
GC: Gastric cancer
HG-ESS: High Grade Endometrial stromal sarcomas
HL: Histiocytic Lymphoma
HLA: Human leukocyte antigen
iPSC: Induced pluripotent stem cell
LA: Lung adenocarcinoma
LC: Liver cancer
LG-ESS: Low Grade Endometrial stromal sarcomas
LLM: Large language model
MASC: Mammary analogue secretory carcinoma
MC: Mucoepidermoid carcinoma
MM: Malignant melanoma
MPN: Myeloproliferative neoplasm
NC: NUT carcinoma
NGS: Next-generation sequencing
NHL: Non-Hodgkin’s Lymphoma
NSCLC: Non-small cell lung cancer
OC: Ovarian cancer
OS: Overall survival
PA: Pilocytic Astrocytoma
PC: Prostate Cancer
PPA: Pulmonary pleomorphic adenoma
PPMS: Primary Pulmonary Myxoid Sarcoma
PROTAC: Proteolysis-targeting chimera
PTC: Papillary thyroid carcinoma
RMS: Rhabdomyosarcoma
RNA-seq: RNA sequencing
RT-PCR: Reverse transcription polymerase chain reaction
SBC: Secretory Breast Carcinoma
scRNA-seq: Single-cell RNA sequencing
SS: Stromal Sarcoma
TALEN: Transcription activator-like effector nuclease
T-ALL: T-cell acute lymphoblastic leukemia
T-LBL: T-cell lymphoblastic lymphoma
TC: Thyroid cancer
WGS: Whole-genome sequencing
ZFNs: Zinc-finger nucleases
